# Different major effects regulating productivity dynamics at different succession stages of monsoon evergreen broad-leaved forests

**DOI:** 10.3389/fpls.2026.1794690

**Published:** 2026-04-07

**Authors:** Lifan Wang, Lang Liu, Ruiguang Shang, Jiayan Shen, Wande Liu

**Affiliations:** 1 Institute of Highland Forest Science, Chinese Academy of Forestry, Kunming, China; 2 Pu’er Forest Ecosystem Research Station, National Forestry and Grassland Administration of China, Pu’er, China; 3 Pu’er Forest Ecosystem Observation and Research Station of Yunnan Province, Pu’er, China

**Keywords:** monsoon evergreen broad-leaved forest, productivity, taxonomic diversity, functional diversity, phylogenetic diversity, succession

## Abstract

**Introduction:**

Biodiversity has a significant impact on the formation and maintenance of ecosystem functions, and its role changes dynamically along with the ecological succession process. However, the relative contributions of biodiversity across different dimensions to ecosystem functions, as well as the patterns of change in these contributions during succession processes, remain unclear.

**Methods:**

This study selected four succession stages of monsoon evergreen broad-leaved forests (early succession, early-to-mid succession, mid-to-late succession, and late succession) as research subjects. Based on vegetation survey data, we calculated three biodiversity metrics (taxonomic diversity, functional diversity, phylogenetic diversity) and functional composition. Using forest productivity as an indicator of ecosystem function, we employed structural equation modeling to analyze how biodiversity across different dimensions at various succession stages influences productivity. This investigation explores how the relative contributions of selection effects, niche complementarity, and mass ratio effects to productivity change throughout forest succession.

**Results:**

The results indicate that: (1) As succession progresses, species richness, functional dispersion, and the phylogenetic PD index exhibit a unimodal pattern. Functional traits predominantly reflect resource acquisition in the early stages, shifting towards conservation in the later stages. Productivity shows fluctuating trends(P < 0.05); (2) The relationship between biodiversity and productivity changes with succession, showing an overall downward trend; (3) In the early succession stage, productivity is primarily driven by the mass ratio effect. During the middle to early succession stage, it is jointly driven by the selection effect and the niche complementarity effect. In the middle to late succession stage, it is mainly driven by the complementarity effect. There is no significant correlation between the late succession stage and biodiversity. Additionally, the local environment also has a significant impact on productivity.

**Discussion:**

The research findings are expected to provide scientific evidence for understanding the formation and maintenance patterns of monsoon evergreen broad-leaved forest ecosystem functions.

## 1 Introduction

Monsoon evergreen broad-leaved forest is one of the typical zonal vegetation types in the southwest region, widely distributed across the southwest and south China regions (Zhu et al., 2019), as the core vehicle for supplying regional ecosystem services, its structural stability and functional sustainability play a vital role in maintaining the ecological balance of subtropical forests (Zhou et al., 2013). Natural succession is the core process driving the dynamic changes within monsoon evergreen broad-leaved forest communities. This process is lengthy and complex, typically accompanied by significant continuous shifts in the community’s species composition, functional traits, and spatial distribution (Kahmen and Poschlod, 2004; Tang, 2010; Chen W. et al., 2025). Community productivity, as a core indicator of ecosystem function, and its relationship with biodiversity constitute a key research topic in ecology (Loreau and Hector, 2001; Mori et al., 2017; Luo et al., 2019). The continuous temporal dynamics of natural succession processes provide an ideal model for studying the evolution of the relationship between biodiversity and productivity. By analyzing community characteristics across different succession stages, we can gain deeper insights into the dynamic patterns governing the relationship between biodiversity and productivity.

The regulatory role of biodiversity on productivity is fundamental to understanding the mechanisms that maintain ecosystem function. The relationship between taxonomic diversity and productivity is highly dependent on scale. Studies at the global scale consistently reveal a clear positive correlation between species diversity and ecosystem function (Liang et al., 2016), however, at the local scale, the relationship exhibits significant uncertainty, potentially showing positive correlation, negative correlation, or a unimodal relationship (Van Der Plas, 2019). This discrepancy indicates that traditional taxonomic diversity metrics cannot comprehensively assess a community’s biodiversity level. Communities may harbor redundant species with similar or overlapping niches, which do not genuinely enhance the ecosystem’s efficiency in utilizing resources such as light, water, and nutrients. Instead, they may intensify interspecific competition (Laughlin and McGill, 2024). Therefore, it is necessary to integrate the multidimensional characteristics of biodiversity and comprehensively consider the synergistic effects of biodiversity across different dimensions on ecosystem functions in order to more accurately elucidate the underlying mechanisms governing the relationship between biodiversity and productivity.

Within the theoretical framework analyzing the relationship between biodiversity and productivity, the niche complementarity effects hypothesis and the mass ratio effects hypothesis represent two core and non-conflicting explanatory mechanisms (Hao et al., 2020; Chen C. et al., 2025). The niche complementary effects hypothesis, grounded in niche differentiation theory, posits that different species within a community reduce resource competition losses through differentiated resource utilization and ecological niche complementarity. This differentiation in resource use significantly enhances the overall resource utilization efficiency of the community, thereby driving increased productivity (Tilman et al., 1997). The mass ratio effect hypothesis focuses on the collective contribution of species functional traits, positing that community productivity depends on the weighted average of all species’ functional traits within the community. That is, the mean value of functional traits determines the functional potential (Grime, 1998). Additionally, selection effects may influence the relationship between the two. As species richness increases, faster-growing and more competitive species appear more frequently in communities. At this point, the impact of functional diversity on productivity may be significantly lower than that of taxonomic diversity. That is, productivity gains rely more on the selection effects of dominant species rather than the complementary interactions among species (Hooper et al., 2005). The gradual shifts in biodiversity and environmental factors during forest succession may dynamically alter the relative contributions of these three ecological mechanisms. Currently, the relative contributions of these three mechanisms to the relationship between forest biodiversity and productivity as succession progresses require further validation.

At the same time, the relationship between biodiversity and productivity is influenced by both biotic and abiotic factors. Among abiotic factors, environmental elements such as climate, soil, and topography not only directly affect the level of forest productivity (Liu et al., 2018; Perry and Oetter, 2024), additionally, by regulating hydrothermal conditions and nutrient supply, these factors influence species distribution, growth, and competitive processes. Through their impact on biodiversity, they indirectly affect the interactions between biodiversity and productivity (Zhang et al., 2024; Yang T. et al., 2025). The relationship between biodiversity and productivity exhibits significant variation with changes in environmental conditions. Under favorable environmental conditions, mechanisms such as species complementarity and dominant species selection typically contribute to enhanced productivity (Pretzsch et al., 2013; Murphy et al., 2015). Therefore, in-depth analysis of the regulatory mechanisms by which environmental factors influence the relationship between biodiversity and productivity is crucial for understanding and predicting changes in forest ecosystem functions.

This study examines fixed monitoring plots at different successional stages in the Pu’er region. Based on vegetation survey data collected in 2014 and 2024, and integrating phylogenetic and functional trait data of plants, structural equation modeling was employed to examine the relationship between biodiversity and productivity across various successional stages. The following questions will be addressed: (1)How do biodiversity and productivity change with succession? (2) What are the principal processes driving changes in productivity? The research findings are expected to provide scientific support and theoretical basis for the conservation of biodiversity in monsoon evergreen broad-leaved forests, the sustainable management of ecosystems, and the maintenance of regional ecological security.

## 2 Materials and methods

### 2.1 Study area

**Figure 1:**
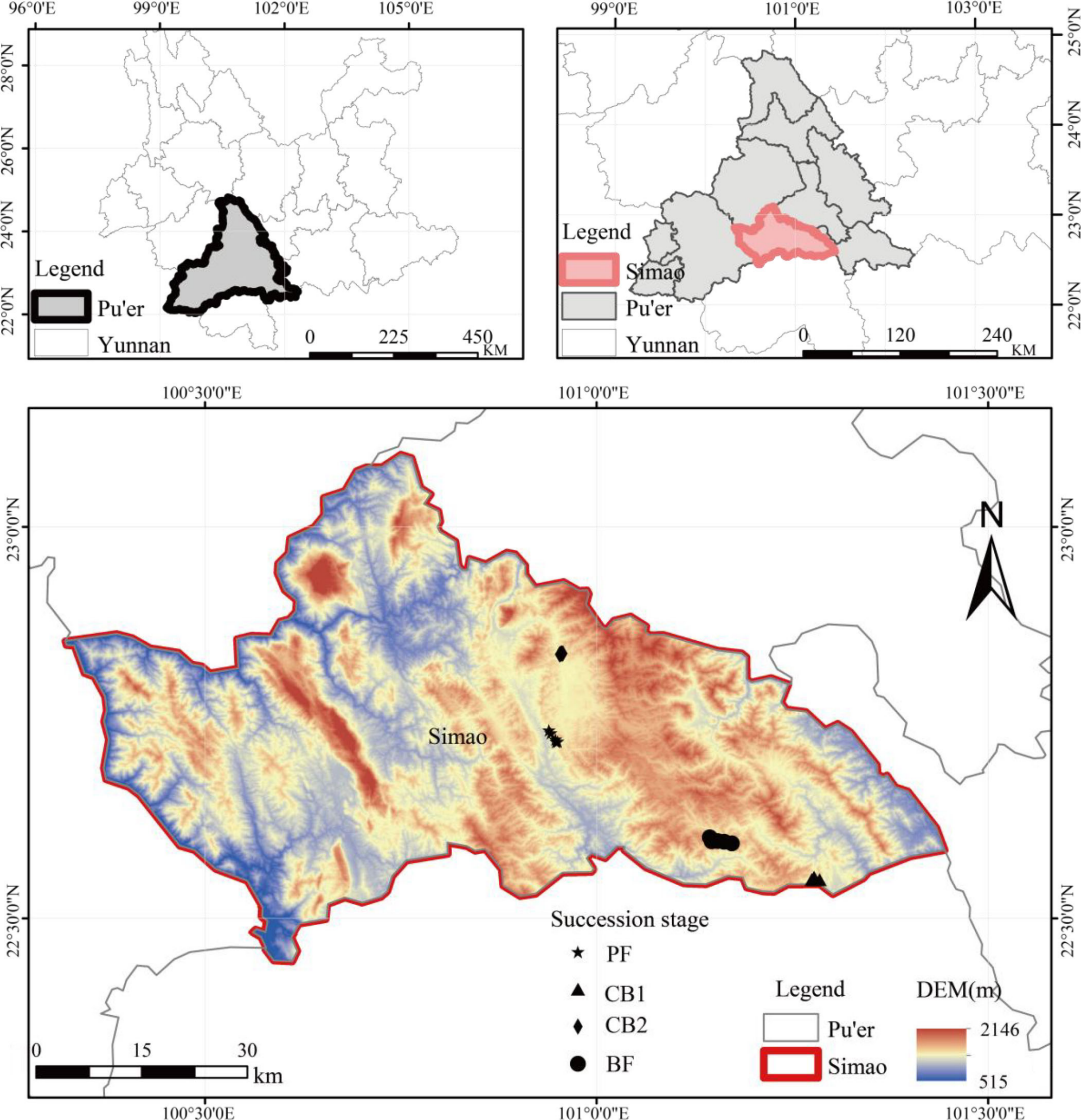
Location of the study area and distribution of sample plots. PF, early succession; CB1, early-to-mid succession; CB2, mid-to-late succession; BF, latexsuccession.

The study area is located in Simao District, Pu’er City, Yunnan Province (22°27′˜23°06′N, 100°19′˜101°27′E), elevation range from 376 m to 3,306 m. This region falls within the subtropical plateau humid monsoon climate zone, with an average annual temperature of 17.8°C and an average annual precipitation of 1,547.6 mm (Qiao et al., 2024). Rainfall during the rainy season (July to September) accounts for approximately 85% of the annual total, and the predominant soil type is laterite (Zhang et al., 2023) (**Figure 1**).

### 2.2 Plot establishment and field surveys

Sample plots were established in 2014, and the initial survey was conducted, including four successional stages: early successional stage (Pinus kesiya var. langbianensis forest), early-middle successional stage (coniferous-broadleaved mixed forest), middle-late successional stage (mature coniferous-broadleaved mixed forest), and late successional stage (monsoon evergreen broad-leaved forest). A total of 20 sample plots were set up, with 5 plots in each successional stage, established in accordance with CTFS standards (Condit, 1995). Each 60m×60m sample plot was divided into 9 20m×20m subplots, and a community survey was carried out. Every woody plant individual in the subplots was measured by diameter at breast height (DBH), soil samples were collected simultaneously, and information such as longitude and latitude was recorded. A re-survey of the above sample plots was conducted in 2024.

### 2.3 Soil data collection

After processing, soil samples were primarily analyzed for soil pH (pH), soil organic matter (SOM, g/kg), soil total nitrogen (TN, g/kg), soil total phosphorus (TP, g/kg), soil total potassium (TK, g/kg), soil available nitrogen (HN, mg/kg), soil available phosphorus (AP, mg/kg), soil available potassium (AK, mg/kg)(**Table 1**). Soil pH was determined by the potentiometric method, soil organic matter (SOM) by the potassium dichromate method with added heat capacity, and soil total nitrogen (TN), soil total phosphorus (TP), and soil total potassium (TK) were measured using a Kjeldahl nitrogen analyzer, potassium hydroxide fusion flame photometry, and molybdenum blue photometry, respectively. Soil available nitrogen (HN), available phosphorus (AP), and available potassium (AK) were determined using the alkali leaching diffusion method, molybdenum blue photometry, and ammonium acetate extraction flame photometry, respectively (Gao et al., 2025).

**Table 1:** Analysis of differences in soil nutrients during the succession of monsoon evergreen broad-leaved forests.

Succession stage	PF	CB1	CB2	BF
pH	4.92 ± 0.18a	4.24 ± 0.20c	4.82 ± 0.25a	4.63 ± 0.18b
OM/g.kg^-1^	25.67 ± 11.93d	44.51 ± 12.37c	61.50 ± 16.00b	84.74 ± 28.00a
TN/g.kg^-1^	0.08 ± 0.03c	0.16 ± 0.06b	0.19 ± 0.04b	0.28 ± 0.07a
HN/mg.kg^-1^	83.26 ± 30.05d	128.30 ± 42.94c	247.30 ± 55.26b	337.00 ± 72.90a
TP/g.kg^-1^	0.02 ± 0.01c	0.02 ± 0.01c	0.03 ± 0.01b	0.04 ± 0.01a
AP/mg.kg^-1^	2.45 ± 1.18c	10.13 ± 2.97b	10.60 ± 3.95b	19.29 ± 4.94a
TK/g.kg^-1^	0.99 ± 0.52a	0.41 ± 0.26b	0.39 ± 0.14b	0.47 ± 0.22b
AK/mg.kg^-1^	203.10 ± 80.45a	106.20 ± 44.96c	165.90 ± 76.93ab	143.60 ± 30.93b

### 2.4 Functional trait collection

The main measured plant functional traits include leaf area (LA), specific leaf area (SLA), leaf dry matter content (LDMC), wood density (WD), leaf organic carbon content (LC), leaf nitrogen content (LN), and leaf phosphorus content (LP) (Yang R. et al., 2025). Twenty mature leaves were selected for each species; after collection, the leaves were taken indoors to immediately determine indicators such as leaf length, width, area, and thickness. After drying to a constant weight for dry weight measurement, the leaves of the same species were mixed, crushed, ground, and sieved for the determination of carbon (C), nitrogen (N), and phosphorus (P) contents in the leaves.

Wood density was obtained through three methods. For species with individuals having a diameter at breast height (DBH) greater than 5 cm, wood density was measured by drilling annual ring strips with a tree growth cone. For species where all individuals have a DBH less than 5 cm, 2-year-old branches were collected, and wood density was determined using a densitometer (Xu T. et al., 2023).

### 2.5 Data processing and analysis

#### 2.5.1 Species diversity

This study selected the species richness index (SR) to characterize the taxonomic diversity level of the sample plots. Calculations were performed using the vegan package in R software (Zhao et al., 2025), see Equation 1:

(1) SR=N

in the formula, N denotes the number of species within the community.

#### 2.5.2 Functional diversity

This study employs the functional dispersion index (FDis) to quantify functional diversity. This index calculates the average distance of all species within a community from the center of the trait space, serving to measure the degree of dispersion in species distribution across functional trait space. It more effectively reflects how community species occupy the ecological niche space. Perform the calculation using the FD package (Mason et al., 2005), see Equation 2:

(2) $$FDis=\frac{\sum_{i=1}^{N}d_{i}\times w_{i}}{\sum_{i=1}^{N}w_{i}}$$

in the formula, *N* denotes the number of species within the community, ${\text{d}}_{\text{i}}$ represents the distance from species i to the center of the trait space, and ${\text{w}}_{\text{i}}$ indicates the biomass proportion of species i within the community.

#### 2.5.3 Phylogenetic diversity

Phylogenetic diversity is quantified using Faith’s phylogenetic diversity index (Faith’s PD). Faith system diversity describes the sum of evolutionary differences among all species within a community. Perform the calculation using the picante package (Chen J. et al., 2025), see Equation 3:

(3) Faith's PD=∑i=1Nlengthi

in the formula, *N* denotes the number of species within the plot, ${\text{length}}_{\text{i}}$denotes the evolutionary branch length of species i in the phylogenetic tree.

#### 2.5.4 Community weighted mean

Based on functional trait data of plant species, calculate the weighted mean functional traits (CWM) for communities at different successional stages and compare functional trait differences among communities. Perform the calculation using the FD package (Violle et al., 2007), see Equation 4:

(4) $$CWM=\sum_{i=1}^{N}P_{i}\times Trait_{i}$$

in the formula, N represents the total number of species in the community, CWM denotes the weighted average of community functional traits, ${\text{P}}_{\text{i}}$ indicates the relative abundance of species i within the community, and ${\text{Triat}}_{\text{i}}$ signifies the average functional trait value of species i.

#### 2.5.5 Productivity

The biomass of tree species within the plot during two vegetation surveys was calculated using allometric growth equations (**Table 2**), productivity is measured by the sum of all individual above-ground biomass increments from 2014 to 2024. This productivity is then divided by time and sample plot area to yield the average annual above-ground productivity per hectare (hereafter referred to as productivity) (Van Der Sande et al., 2018).

**Table 2:** Allometric growth equations for above-ground biomass of major tree species in the study area.

Tree species	Organ	Allometric growth equation
*Castanopsis echidnocarpa*	Stem	W=9.7566 + 0.014877×DBH³
	Branch	W=1.4497 + 0.0069051×DBH³
	Leaf	W=0.03015231×(-0.262+DBH)²
*Pinus kesiya* var. *langbianensis*	Stem	W=0.01218×(DBH²H)×0.9998 + 0.02340×DBH^2.4247^
	Branch	W=0.00028×(DBH²H)×1.2526
	Leaf	W=DBH²H/(0.023×DBH²H+1967.57)
Other broad-leaved species	Stem	W=0.080443×DBH^2.5142^
	Branch	W=0.00000029416×(7.5074+DBH)^5^
	Leaf	W=0.8442×exp(0.1214×DBH)-0.9650

### 2.6 Statistical analysis

First, a one-way analysis of variance (ANOVA) was conducted to examine differences in biodiversity and productivity across various succession stages. To further elucidate the direct or indirect effects of biotic and abiotic factors on productivity, a partial least squares structural equation model (PLS-SEM) was constructed using the “plspm” package in R 4.3.3 software (Xu L. et al., 2023). This model enables systematic investigation of complex relationships between observed variables and latent variables. Compared to other structural models, the Partial Least Squares Structural Equation Modeling approach requires fewer samples and does not necessitate data to follow a normal distribution (Sarstedt et al., 2020). First, perform spearman correlation analysis using the “linkET” package in R 4.3.3 software to screen significant factors as observed variables. Significant factors were then grouped into five latent variables: taxonomic diversity, functional diversity, phylogenetic diversity, community-weighted mean of functional traits, and environmental factors. After excluding factors with standard loadings< 0.7, the influence of each latent variable on productivity was analyzed. Next, create a complex initial model to display all important variables from a plausible interaction pathway. Finally, remove insignificant variables from the model to obtain the model with the highest fit. Evaluate the fit of the structural equation model using the Goodness-of-fit (GOF) index.

## 3 Results

### 3.1 Changes in biodiversity and productivity during succession

**Figure 2:**
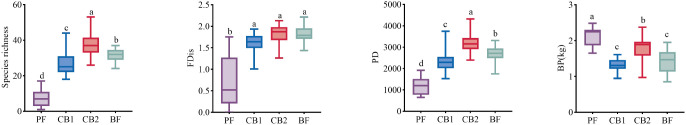
Dynamic changes in biodiversity and productivity with succession. PF, early succession; CB1, early-to-mid succession; CB2, mid-to-late succession; BF, late succession. Different lowercase letters indicate significant differences between different successional stages (*P* < 0.05).

The results of one-way ANOVA indicate that species richness, functional dispersion index, and phylogenetic PD index exhibit a “unimodal” trend of initial increase followed by decline throughout succession, with peaks occurring during the mid-to-late succession stages (**Figure 2**). Productivity fluctuates throughout succession, reaching its highest levels during the early succession phase.

### 3.2 Changes in functional traits during succession

**Figure 3:**
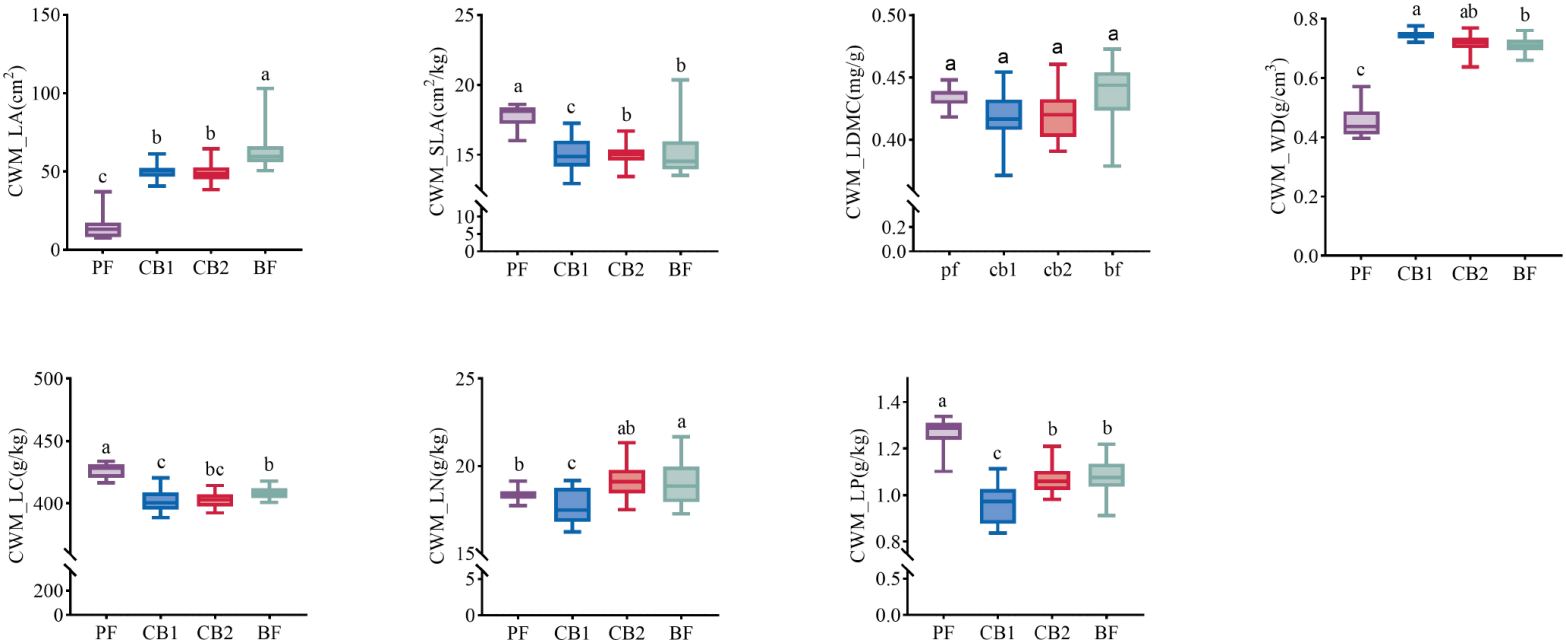
Dynamic changes in the weighted mean of community functional traits with succession. PF, early succession; CB1, early-to-mid succession; CB2, mid-to-late succession; BF, late succession. Different lowercase letters indicate significant differences between different successional stages (*P* < 0.05).

All functional traits, except the community-weighted mean of leaf dry matter content (CWM_LDMC), have significant differences (P < 0.05) across different succession stages (**Figure 3**). Specifically, as succession progressed, the LA showed an overall increasing trend, while the LDMC exhibited a decreasing trend. The community-weighted mean of wood density first increased and then decreased, reaching its maximum value in the early to mid-successional stages. The LC, LN, LP showed an overall trend of first decreasing and then increasing. During the early stages of community succession, functional traits are primarily resource-acquisition oriented; in the later stages, they shift toward a more conservative type.

### 3.3 Structural equation modelling

**Figure 4:**
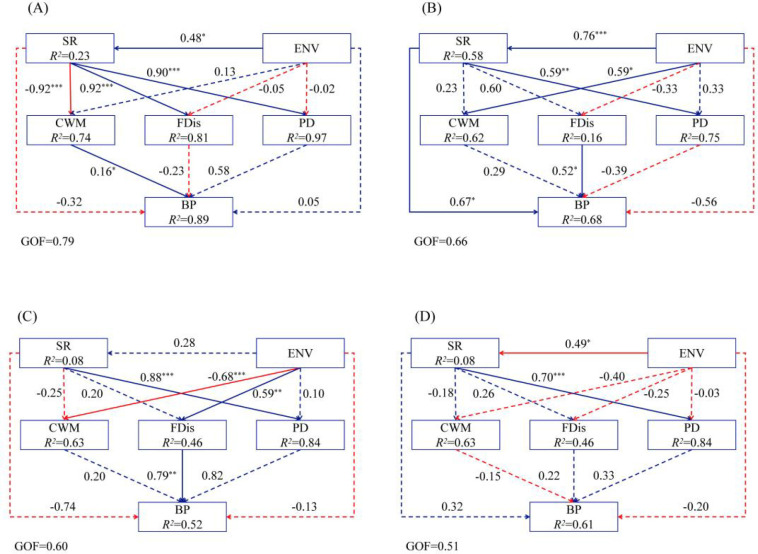
Integrated effects and effect values of soil factors, species richness, functional dispersion, functional composition, and phylogenetic PD index on forest stand productivity across different successional stages. The blue line represents positive effects, while the red line denotes negative effects. Solid lines indicate significant effects, while dashed lines denote non-significant effects. * indicates significance at *P<* 0.05, ** indicates significance at *P* < 0.01, *** indicates significance at *P* < 0.001. ENV, Environmental factors; SR, Species richness; CWM, Functional composition; FDis, Functional Dispersion; PD, Phylogenetic PD index; BP, Productivity. **(A)** early succession; **(B)** early-to-mid succession; **(C)** mid-to-late succession; **(D)** late succession.

The structural equation model demonstrated overall good goodness-of-fit (GOF > 0.5). As succession progressed, the relationship between biodiversity across different dimensions and productivity underwent significant changes. During the Pinus kesiya var. langbianensis forest stage (early succession phase, **Figure 4**A), environmental factors and biodiversity together explained 89% of productivity variation, with productivity being significantly and positively influenced by functional composition (r = 0.16). For mixed coniferous and broad-leaved forests (early to mid-successional stage, **Figure 4B**), environmental factors and biodiversity together explained 68% of productivity variation. Productivity was significantly and positively influenced by functional dispersion (r = 0.52) and species richness (r = 0.67). For old-growth mixed coniferous and broad-leaved forests (in the mid-to-late succession stage, **Figure 4C**), environmental factors and biodiversity together explained 52% of productivity variation. Productivity was primarily influenced by a significantly positive effect of functional dispersion (r = 0.79). For monsoon evergreen broad-leaved forests (early to mid-successional stages, **Figure 4D**), environmental factors and biodiversity together explained 61% of productivity variation, with no single factor significantly influencing productivity.

## 4 Discussion

### 4.1 Patterns of biodiversity and productivity changes during succession

This study found that species richness, functional dispersion index, and phylogenetic PD index exhibited high consistency “unimodal pattern” trends that first increased and then decreased as succession progressed. The peaks were concentrated in the middle to late stages of succession. This dynamic variation is closely associated with resource allocation, interspecies interactions, and changes in community structure during the succession process of forest ecosystems. During the early stages of succession, species possessing rapid foliar economic traits dominate, resulting in a simple stand structure (Broadbent et al., 2014), The community is dominated by the coniferous species *Pinus kesiya* var. *langbianensis* as the absolute dominant species. Spatial heterogeneity in resources such as understorey light, moisture, and nutrients is relatively low, allowing pioneer species to rapidly spread and grow, occupying the majority of ecological niches. The allelopathic effects of coniferous species inhibit the growth of broad-leaved species (Wang et al., 2025), creating a density-limiting effect that results in lower species richness. *Pinus kesiya* var. *langbianensis* exhibit a tendency towards morphological convergence, with species predominantly concentrated within a few closely related groups such as the Pinaceae family, showing limited functional and phylogenetic differentiation. During the early-to-mid succession stage of mixed coniferous and broad-leaved forests, the community establishes a stratified light environment through varying canopy heights. This accommodates the growth requirements of shade-tolerant, neutral, and light-demanding species. Concurrently, the accelerated decomposition rate of broad-leaved litter enhances soil nutrient cycling and elevates readily available nitrogen and phosphorus levels. This significantly increases habitat heterogeneity (Getaneh et al., 2022); at this stage, the proportion of conifers and broad-leaved trees within the community gradually approaches equilibrium. While exhibiting a degree of competition for resources, they also achieve complementarity through ecological niche differentiation. This creates conditions conducive to the settlement of additional species, propelling species richness into a phase of rapid ascent. The diversification of functional traits among species significantly increased the functional dispersion index. The inclusion of broad-leaved tree species broadened the phylogenetic spectrum of the community. The aggregation of species from different families and genera drove a concurrent rise in the phylogenetic PD index, which continued to increase throughout the early and middle stages of succession. As succession progressed into its mid-to-late stages, habitat heterogeneity and resource complementarity effects intensified further, ultimately leading to simultaneous peaks in species richness, functional dispersion index, and phylogenetic PD index. As succession progresses to the mature stage of monsoon evergreen broad-leaved forests, the canopy layer is dominated by evergreen broad-leaved species such as *Fagaceae* and *Lauraceae*, whose competitive advantage in light and deep soil nutrients significantly surpasses that of other species. Concurrently, the competitive exclusion exerted by dominant broad-leaved tree species suppresses the survival of certain understorey plants and less competitive tree species, constricting the functional space available to other species. Distant relatives are also eliminated due to unsuccessful resource competition, progressively limiting the space for species survival and development within the habitat. At this stage, the dominant evergreen broad-leaved tree species within the community further consolidated their position. Their competitive advantage exerted a significant influence on the community structure, leading to a slight decline in species richness. Functional dispersion decreased due to heightened trait convergence, while the phylogenetic PD index concurrently declined as the phylogenetic range narrowed. Productivity also exhibits significant variations across different stages of succession; in this study, productivity fluctuations were observed. As a sun-loving coniferous species, *Pinus kesiya* var. *langbianensis* exhibits high photosynthetic efficiency during its juvenile and sub-mature stages, with resource allocation favouring above-ground growth. Consequently, productivity reaches its peak. Although mature forests in the middle to late stages of succession exhibit higher respiratory consumption than younger stands, their prolonged evolutionary process has fostered a more complex community structure. This enables more stable decomposition of litter and nutrient cycling beneath the canopy (Getaneh et al., 2022). Moreover, the substantial biomass base of individual trees ensures that net productivity remains sustained at levels exceeding those observed in the early to middle stages of succession, This is consistent with the conclusion proposed by Fang Jingyun et al. that the rate of decline in productivity of old-growth forests slows as community stability increases (Fang et al., 1996). Monsoon evergreen broad-leaved forests represent the regional climax vegetation type, characterised predominantly by species adopting conservative strategies. Resources are directed towards maintaining high biomass and stability, resulting in relatively low forest productivity.

### 4.2 The relationship between biodiversity and productivity and its patterns of change during succession

The findings of this study indicate that the relationship between biodiversity and productivity exhibits significant variations throughout forest succession. During the early succession phase, the mass ratio effect serves as the primary mechanism influencing productivity. In the middle to early succession stages, both the selection effect and the niche complementarity effect jointly impact productivity. The role of niche complementarity becomes more pronounced in the middle to late succession phases. By the late succession stage, no significant correlation with biodiversity is observed. During the early succession phase, the *Pinus kesiya* var. *langbianensis* forest serves as a pioneer community characterised by high resource heterogeneity and relatively weak competition among species. Environmental selection pressures drive functional trait convergence within the community, with newly introduced species exhibiting high similarity to *Pinus kesiya* var. *langbianensis* in key traits. This process enables species possessing highly efficient resource utilisation traits to become dominant. This study found that species richness primarily exerts an indirect influence on productivity through its effects on functional traits, with the mass-ratio effect hypothesis playing a significant role at this stage. Species possessing advantageous functional traits dominate productivity changes. We observed higher specific leaf area during the early succession phase (**Figure 4b**), largely consistent with our expectations. This is because primary productivity during this phase stems predominantly from dominant tree species such as *Pinus kesiya* var. *langbianensis*. These species exhibit large individual size, high abundance, small leaf area, large specific leaf area, and uniform distribution. This finding aligns with the conclusions drawn by Matsuo et al. in their study of succession in Mexican tropical secondary forests, which revealed that pioneer tree species exhibiting functional advantages during the early succession phase achieve higher relative biomass growth rates by enhancing light capture efficiency, thereby providing the primary productivity for the community (Matsuo et al., 2024). Functional traits of species are linked to resource niches, exerting direct or indirect influences on their adaptive development and performance. Moreover, functional traits more effectively reflect niche differentiation among species, offering deeper insights into interspecific dynamics (Funk et al., 2016; Yang et al., 2016). As forest succession progresses, species diversity progressively alters species composition, not only by increasing species richness but also by modifying species evenness, thereby reshaping the range and distribution of functional traits within the community. Whilst taxonomic diversity facilitates functional differentiation, it may fail to fully capture key functional trait differences between species (Flynn et al., 2011; Barabás et al., 2022; Gu et al., 2025). During this successional stage, selection pressure primarily enhances community productivity by favouring dominant species with high photosynthetic efficiency and resource utilisation capacity. These dominant species, owing to their functional advantages, become the primary drivers of biomass accumulation within the community; complementary effects, meanwhile, rely on the differentiation of functional traits between species to achieve efficient resource integration. This complementary resource utilisation effectively reduces competition losses between species, significantly enhancing the overall resource efficiency of the community. The two mechanisms do not operate in isolation but exhibit synergistic characteristics, jointly driving the accumulation of forest productivity. These findings are highly consistent with the conclusions drawn by Xu et al. in their study of subtropical forests in China (Xu et al., 2024). This further validates that the associative mechanism between biodiversity and ecosystem productivity in forest ecosystems holds broad applicability and key ecological significance. During the middle and late stages of succession, the mechanisms driving ecosystem productivity undergo significant shifts compared to the early and middle stages.The influence of selection effects becomes negligible at this stage, with species richness showing no significant correlation with productivity. In stark contrast, the path coefficient linking functional dispersion to productivity increases markedly, indicating that complementary effects have emerged as the primary mechanism driving ecosystem productivity. At this stage, the community structure tends towards complexity and stability. Most species have developed adaptive capabilities enabling their survival within the existing environment, leading to a weakening of the screening effect exerted by a single dominant species. Consequently, a simple increase in species numbers no longer directly translates into enhanced productivity (Isbell et al., 2009). Instead, through prolonged ecological niche differentiation and species coexistence, highly complementary resource utilisation strategies have emerged within the community. This finding aligns with the results from Deng et al.’s study in China’s subtropical forest biodiversity experiment, which revealed that during the early to mid-successional stages (forest age< 12 years), selection effects and complementary effects synergistically drove productivity. In the mid to late stages (forest age ≥ 12 years), selection effects dissipated, leaving complementary effects as the sole dominant mechanism (Deng et al., 2025). Bongers et al. also observed similar results based on a decade of data from the Chinese Subtropical Forest Biodiversity Experiment. They found that the impact of functional diversity on forest productivity and the reliability of its predictive power increased with forest age, surpassing the community-weighted average after seven years. Furthermore, the enhancing effect of functional diversity on productivity was not constrained by species pool size (Bongers et al., 2021). In the late succession stage, mature monsoon evergreen broad-leaved forests constitute the climax community, exhibiting stable structural and functional characteristics. Species competition and resource allocation reach equilibrium, with carbon accumulation constrained by environmental carrying capacity. Plants redirect greater resources towards reproduction, defence mechanisms, and energy storage to maintain population stability, resulting in a decline and stabilisation of carbon accumulation rates. At this stage, biodiversity shows no significant correlation with productivity, as the function of diversity shifts from driving productivity to maintaining system stability: balanced networks form between species, mitigating fluctuations in individual species. Moreover, newly introduced species often exhibit functional redundancy, making it difficult to enhance resource utilisation efficiency and thus failing to significantly drive productivity (Lautenbach et al., 2012). However, this study has certain limitations. Although the 10-year monitoring period is comparable to that of the aforementioned studies, it still fails to fully capture the entire process of forest succession. The research area is limited to Pu’er only, resulting in limited generalizability of the findings. Additionally, the study focuses on woody plants and aboveground productivity, excluding understory organisms, soil microorganisms and belowground ecological processes, which leads to an incomplete analytical dimension.

## 5 Conclusion

This study focused on four successional stages of the monsoon evergreen broad-leaved forest in Pu’er, Yunnan Province, and analyzed the dynamic correlation between biodiversity and aboveground productivity based on a 10-year monitoring dataset from 2014 to 2024. The results showed that biodiversity exhibited a unimodal pattern with forest succession, and community functional traits shifted from resource-acquisitive to conservative types. The correlation between biodiversity and productivity gradually weakened during the successional process, with the driving mechanisms showing a stage-specific succession; in addition, local environmental factors remained an important influencing factor throughout all stages.

Future research will focus on expanding the spatiotemporal scale by conducting cross-regional long-term monitoring and refining the division of successional stages, and improving the research system by incorporating underground functional traits, understory organisms and soil microorganisms. These efforts are expected to provide scientific support for forest ecological restoration, biodiversity conservation and adaptive management in the context of climate change.

## Data Availability

The raw data supporting the conclusions of this article will be made available by the authors, without undue reservation.
